# Integrative proteomics reveals mitochondrial and immune signatures of MLH1 exon 13 deletion in Lynch syndrome–associated colorectal cancer

**DOI:** 10.3389/fmolb.2025.1722111

**Published:** 2025-12-17

**Authors:** Chen Chang, Yue Cao, Bin Zhang, Jingli Chen, Lin Chen, Wensheng Li, Guorong Wang

**Affiliations:** 1 Department of Pathology, Shaanxi Provincial People’s Hospital, Xi’an, Shaanxi, China; 2 First Department of General Surgery, Shaanxi Provincial People’s Hospital, Xi’an, Shaanxi, China

**Keywords:** Lynch syndrome, MLH1 large genomic rearrangement, proteomics, oxidative phosphorylation, ribosome biogenesis, immune microenvironment

## Abstract

**Background:**

Lynch syndrome is an inherited cancer predisposition caused by pathogenic variants in mismatch repair (MMR) genes. Large genomic rearrangements (LGRs) in MLH1 are often underestimated due to detection challenges. Functional analyses of specific variants such as MLH1 exon 13 deletion (MLH1-EX13 Del) remain scarce.

**Methods:**

A three-generation Chinese family with Lynch syndrome was investigated. Targeted next-generation sequencing identified MLH1-EX13 Del in the proband, which was validated by qPCR in family members. Cancer patients underwent MMR immunohistochemistry (IHC) and microsatellite instability (MSI) testing. Data-independent acquisition proteomics was performed on four paired tumor and adjacent tissues, followed by Gene Ontology and KEGG enrichment analyses.

**Results:**

Six malignant tumors were diagnosed in the family. All tested carriers harbored MLH1-EX13 Del. IHC showed loss of MLH1 and PMS2, occasionally with focal MLH1 positivity or concurrent MSH2 loss. All tumors tested were MSI-H. Proteomics revealed systemic downregulation of oxidative phosphorylation across mitochondrial respiratory complexes, whereas ribosome biogenesis proteins were upregulated, indicating enhanced protein synthesis. Immune pathway analysis revealed activation of neutrophil-mediated immunity and upregulation of inflammatory markers (S100A8/A9, MPO, ELANE), consistent with an inflamed tumor phenotype.

**Conclusion:**

This study provides the first proteomic evidence linking MLH1-EX13 Del to suppressed mitochondrial metabolism and immune activation. These findings highlight metabolic vulnerability and an inflammatory microenvironment as potential therapeutic targets, offering new insights into Lynch syndrome-associated colorectal cancer.

## 1 Introduction

Lynch syndrome (LS) is the most common hereditary colorectal cancer (CRC) syndrome, accounting for approximately 2%–3% of all CRC cases (Abu-Ghazaleh et al., 2022). Its pathogenesis primarily results from germline pathogenic variants in key genes of the DNA MMR pathway, including MLH1, MSH2, MSH6, and PMS2. MMR deficiency leads to MSI, which not only serves as a hallmark molecular feature of LS-associated tumors but also represents an important prognostic and predictive biomarker, particularly for identifying patients who are likely to respond favorably to immune checkpoint inhibitors (ICIs) (Idos et al., 1993).

Among the pathogenic genes associated with LS, *MLH1* variants are the most prevalent, accounting for approximately 40%–50% of LS families in various studies (Peltomaki et al., 2023). In addition to common point mutations and small insertions/deletions, LGRs involving one or more exons represent an important pathogenic mechanism. In certain LS cohorts, such rearrangements account for 10%–20% or more of pathogenic variants in *MLH1* and *MSH2*, with the exact proportion varying across populations and detection methods (Fanale et al., 2022). Due to the limited sensitivity of routine NGS in detecting structural variants, these LGRs are often underestimated or missed in clinical testing (Witt et al., 2023). *MLH1*-EX13 Del is a rare type of LGR, with most reports being isolated cases or small pedigrees (McVety et al., 2006; Chong et al., 2009; van der Klift et al., 2005; Liu et al., 1995). Although these studies suggest its pathogenicity, the detailed clinical phenotype spectrum, molecular pathogenic mechanisms, and population distribution of this variant remain less well characterized compared to point mutations or other common LGRs (Huang et al., 2022).

Although the pathway from *MLH1* gene alterations to the dMMR/MSI-H phenotype has been largely elucidated, there is still limited knowledge regarding how this upstream genetic event systematically reshapes the tumor proteome, thereby influencing metabolic patterns, invasive potential, and immune microenvironment characteristics. While genomics and transcriptomics provide a molecular blueprint, proteins, as the direct executors of cellular functions, more accurately reflect the functional state of cells and the activity of signaling pathways. Proteomic analysis can comprehensively reveal alterations in metabolic networks, signal transduction regulation, and microenvironmental composition under specific genetic backgrounds, serving as a crucial bridge between genotype and phenotype.

This study was conducted on a three-generation Chinese family with LS, in which the proband was found to harbor a heterozygous large deletion of exon 13 in the MLH1 gene by NGS, and this variant was further validated in multiple family members using qPCR. We systematically collected clinical data, pathological characteristics, and results from IHC and MSI testing. In addition, we performed DIA-based quantitative proteomic analysis, enabling a multi-omics characterization of the complete molecular cascade from the MLH1 germline mutation to functional phenotypes, including metabolic reprogramming and alterations in the immune microenvironment. The aim of this study was to elucidate the molecular mechanisms driven by LGRs of MLH1 in LS and to provide reference evidence for precision treatment and family management in such patients.

## 2 Materials and methods

### 2.1 Study subjects and ethical statement

This study was conducted in strict accordance with the ethical principles of the Declaration of Helsinki and was approved by the Ethics Committee of Shaanxi Provincial People’s Hospital (Approval No.2025R022). Written informed consent was obtained from all participants or their legal guardians after they were fully informed of the study objectives, procedures, and potential risks. The proband (family ID: II-2) was a CRC patient carrying a heterozygous deletion of exon 13 in the MLH1 gene (*MLH1* EX13 Del), identified through targeted NGS. A detailed three-generation pedigree was constructed through comprehensive family history investigation, confirming a hereditary LS family. Cascade screening was subsequently offered to all available family members, and six core members (including the proband) underwent genetic testing. Demographic data, clinical diagnoses (including cancer type, age at onset, histopathological classification, and treatment history), and molecular pathology test results were systematically collected for all participating members. All clinical and pathological data were verified through review of original medical records and diagnostic reports.

### 2.2 Sample collection and DNA extraction

Peripheral blood samples for germline genetic analysis were collected in EDTA anticoagulant tubes. Tumor molecular pathology analyses were performed on archived formalin-fixed, paraffin-embedded (FFPE) tissue blocks, including tumor tissues and paired adjacent normal tissues located more than 5 cm from the tumor margin. All FFPE blocks were reviewed by two pathologists on hematoxylin and eosin (H&E)–stained slides to ensure that the selected tumor areas contained more than 70% tumor cells. Genomic DNA (gDNA) was extracted from peripheral blood and FFPE tissues using the QIAamp DNA Blood Mini Kit and QIAamp DNA FFPE Tissue Kit, respectively (both from QIAGEN, Germany), following the manufacturer’s instructions. DNA quality and purity were assessed using a NanoDrop 2000 spectrophotometer (Thermo Fisher Scientific, United States). Samples meeting quality control criteria (A260/A280 ratio between 1.8 and 2.0) were stored at −80 °C until further use.

### 2.3 Genetic analysis

Genomic DNA (gDNA) from the proband (II-2) was subjected to targeted capture-based NGS using a commercially available multigene panel covering 32 hereditary cancer predisposition genes related to the digestive system (OncoH-Digestive System, Beijing GenePlus Medical Laboratory, China). NGS analysis revealed a heterozygous LGR involving deletion of exon 13 in the MLH1 gene (MLH1 EX13 Del). To validate this deletion and perform genetic tracing within the family, qPCR copy number variation analysis was conducted on gDNA samples from six core family members, including the proband. The qPCR assay was performed on an ABI 7500 Real-Time PCR System (Applied Biosystems, United States), and the relative copy number (RQ) of MLH1 exon 13 was calculated using the comparative quantification method to confirm the heterozygous deletion status.

### 2.4 MMR protein immunohistochemistry and microsatellite instability testing

FFPE tumor tissues from four CRC patients within the family (II-2, III-6, III-11, and III-21) were subjected to IHC analysis for MMR proteins. Staining was performed on a Ventana Benchmark XT automated immunostainer (Roche Diagnostics, Switzerland) using ready-to-use monoclonal antibodies against MLH1 (clone ES05), MSH2 (clone G219-1129), MSH6 (clone SP93), and PMS2 (clone A16-4) (all from Roche Diagnostics). Slides were independently evaluated in a blinded manner by two experienced pathologists. Normal epithelial or stromal cell nuclei within the same section served as internal positive controls. Complete absence of nuclear staining in tumor cells was recorded as loss of expression. The combination pattern of protein loss was used to infer the likely upstream pathogenic gene involved (Samowitz, 2015).

MSI status was assessed using a commercially available MSI detection kit based on fluorescent PCR-capillary electrophoresis (Registration No. 20213400936; Beijing Yuwei Gene Technology Co., Ltd., China). Six mononucleotide repeat loci (BAT-25, BAT-26, NR-21, NR-24, NR-27, and MONO-27) and three reference loci were amplified via multiplex fluorescent PCR. Amplified products were analyzed on an Applied Biosystems 3730xl DNA Analyzer, and electropherograms were interpreted using GeneMapper® software. Loci exhibiting novel allele fragments in tumor DNA compared with matched normal tissue DNA were considered unstable. Samples with instability at two or more mononucleotide loci were classified as MSI-H.

### 2.5 Data-independent acquisition-based quantitative proteomics and bioinformatics analysis

We performed DIA-based quantitative proteomic profiling on four pairs of tumor tissues and matched adjacent normal tissues. Total proteins were extracted using RIPA lysis buffer and quantified by the bicinchoninic acid (BCA) assay, followed by enzymatic digestion with trypsin. Peptide mixtures were separated on a nanoElute ultra-high-performance liquid chromatography system (Bruker) and analyzed using a timsTOF Pro mass spectrometer (Bruker) operated in DIA mode. Raw DIA data were processed with Spectronaut (Biognosys) to construct a spectral library and perform quantitative analysis. Differentially expressed proteins (DEPs) were identified based on the thresholds of |log_2_(T/N)| ≥ 1 and a false discovery rate (FDR) < 0.05. Functional annotation and pathway enrichment analyses of DEPs were conducted using the DAVID and KEGG databases, encompassing GO categorization and KEGG pathway mapping. Visualization was performed in R software (version 4.3.2) using the packages ggplot2 (volcano plots, bar plots, bubble plots), pheatmap (heatmaps), clusterProfiler (enrichment visualization), and ComplexHeatmap (integrated multi-pathway heatmaps).

### 2.6 Proteomic data quality control

To ensure data reproducibility and normalization accuracy, rigorous quality control (QC) procedures were performed after DIA data acquisition. Peptide-level parameters including peptide length distribution, peptide count, and protein coverage were evaluated to verify digestion quality. Quantitative consistency across biological replicates was further assessed using Pearson correlation coefficients (PCC), boxplots of log_2_ intensity, and relative standard deviation (RSD) analysis. Strong inter-sample correlations (r > 0.9) and low RSD values (<0.2 for most quantified proteins) confirmed high reproducibility and quantitative stability. All QC visualizations are presented in Supplementary Figure S1.

### 2.7 Immunohistochemistry

Immunohistochemical staining for MPO was performed on four paired tumor and adjacent normal tissue sections. The primary antibody used was rabbit anti-MPO monoclonal antibody (RAB-0379, MXB Biotechnologies, China, ready-to-use). Tissue sections were deparaffinized, rehydrated, and subjected to antigen retrieval in EDTA buffer (pH 8.0) using microwave heating for 20 min. Endogenous peroxidase activity was blocked with 3% hydrogen peroxide (H_2_O_2_). After cooling, sections were incubated overnight at 4 °C with the primary antibody. Detection was performed using the PV-8000 two-step immunohistochemistry kit (Zhongshan Jinqiao Biotechnology, China), and color development was achieved with a DAB staining kit. Finally, the sections were counterstained with hematoxylin, dehydrated, and mounted with neutral resin. Positive immunoreactivity was indicated by the presence of brown-yellow granular staining in the cytoplasm.

### 2.8 Statistical analysis

All statistical analyses were conducted using R software (version 4.3.2) and GraphPad Prism (version 9.0). Continuous variables were expressed as mean ± standard deviation (SD) or as median with interquartile range (IQR), while categorical variables were summarized as frequencies and percentages. Differences between two groups were assessed using the two-tailed Student’s t-test for normally distributed data or the Mann-Whitney U test for non-normally distributed data. For comparisons among multiple groups, one-way analysis of variance (ANOVA) or the Kruskal–Wallis test was applied as appropriate. All statistical tests were two-sided, and P values <0.05 were considered statistically significant.

## 3 Results

### 3.1 Clinical and genetic characteristics of the family with MLH1 large exon deletion

**FIGURE 1:**
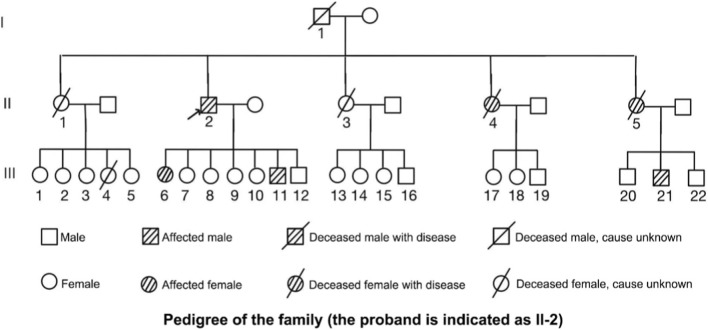
Pedigree of the studied family. The proband is indicated by an arrow (II-2). Squares represent males, circles represent females. Shaded symbols denote affected individuals. Symbols with diagonal slashes indicate deceased individuals, with cause specified where known.

This study involved a three-generation Chinese family in which multiple members were diagnosed with LS–associated gastrointestinal malignancies (Figure 1). The proband (II-2) was diagnosed in 2016 with poorly differentiated adenocarcinoma of the ileocecal region (T3N0M0, stage II) and underwent laparoscopic radical right hemicolectomy, followed by six cycles of FOLFOX4 chemotherapy and subsequent surveillance. To clarify the genetic basis, NGS of the proband’s peripheral blood revealed a large heterozygous deletion encompassing exon 13 of the *MLH1* gene (EX13 Del), annotated in the ClinVar database as a pathogenic variant.

Subsequent germline testing using qPCR in five available family members confirmed the same MLH1 deletion, indicating stable inheritance of this variant within the pedigree. Among carriers, six individuals had developed malignancies, including five cases of CRC and one case of endometrioid carcinoma, with ages at onset ranging from 32 to 63 years (median, 43 years) (Table 1). Primary tumor sites were mainly located in the ileocecal region, sigmoid colon, and rectum. Some patients underwent IHC and MSI testing during clinical management, all showing MLH1/PMS2 loss and MSI-H status (see details in subsequent sections). In addition, cases of pancreatic cancer and rectal cancer were reported in the previous generation, further supporting a strong association between MLH1 deletion and hereditary predisposition to multiple gastrointestinal malignancies within this family.

**TABLE 1:** C linical and molecular characteristics of MLH1 deletion family members.

Family ID	Gender	Age at diagnosis	Cancer type	Tumor location	Histological type	MLH1 status	MSI status	MLH1 IHC	MSH2 IHC	MSH6 IHC	PMS2 IHC	MMR status	Extra CRC tumor
II2	Male	63	Colorectal cancer	Ileocecal region	Poorly differentiated adenocarcinoma	Deletion (+)	MSI-H	-	-	+	-	dMMR	None
II4	Female	40	Rectal cancer									Unknown	Breast cancer
II5	Female	43	Rectal cancer									Unknown	Pancreatic cancer
Ⅲ6	Female	53	Rectal + gynecologic cancer	Mid rectum	Moderately differentiated adenocarcinoma	Deletion (+)	MSI-H	-	+	+	-	dMMR	Endometrial carcinoma (G1)
Ⅲ7	Female	42				Deletion (+)						Unknown	
Ⅲ9	Female	37				Deletion (+)						Unknown	
Ⅲ11	Male	32	Sigmoid colon cancer	Sigmoid colon	Moderately differentiated adenocarcinoma	Deletion (+)	MSI-H	+	+	+	-	dMMR	None
Ⅲ21	Male	40	Rectal cancer	Rectal	Moderately differentiated adenocarcinoma		MSI-H	-	+	+	-	dMMR	None

Abbreviations: dMMR, deficient mismatch repair; pMMR, proficient mismatch repair; MSI, microsatellite instability; IHC, immunohistochemistry.

### 3.2 Immunohistochemical analysis of MMR proteins

**FIGURE 2:**
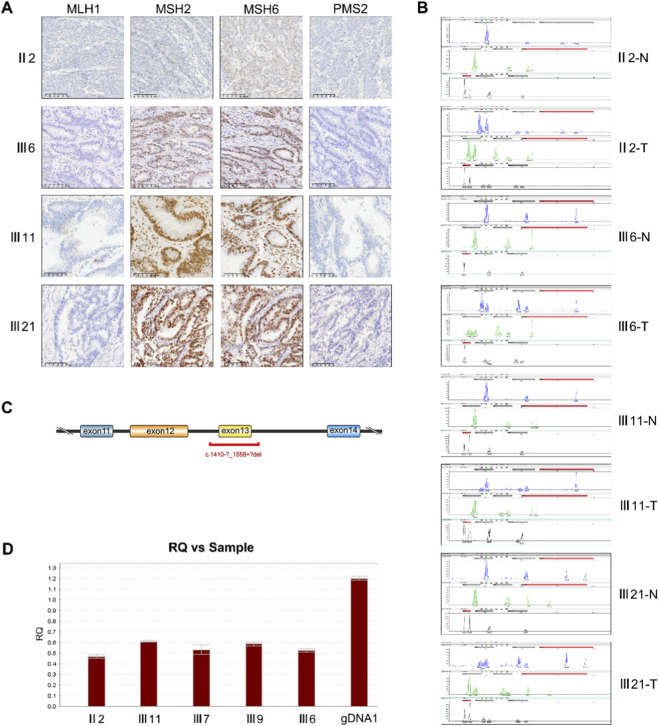
Molecular testing results of tumors from a Lynch syndrome family. **(A)** Immunohistochemical staining of four MMR proteins (MLH1, PMS2, MSH2, MSH6) in tumor tissues from four family members; **(B)** Capillary electrophoresis results of MSI testing. All tumor samples were MSI-H, with corresponding normal tissues showing stable profiles; **(C)** Schematic diagram of the MLH1 gene structure showing exons 11–14. The location of the exon 13 deletion (c.1410-?_1558+?del) is indicated in red to illustrate the affected region; **(D)** Relative quantification (qPCR) of MLH1 exon13. All tested individuals showed ∼50% reduction in RQ compared to the normal control.

To preliminarily screen for MMR deficiency, IHC staining for four key MMR proteins (MLH1, PMS2, MSH2, and MSH6) was performed on tumor tissues from four affected family members (Figure 2A). The results showed that cases Ⅲ6 and Ⅲ21 exhibited complete loss of MLH1 and PMS2 expression with retained MSH2 and MSH6, representing a typical MLH1-type dMMR phenotype. In case Ⅲ11, MLH1 showed focal positivity while PMS2 was completely absent, suggesting a partial dMMR status possibly due to reduced MLH1 expression or instability of PMS2. In contrast, the proband (II-2) demonstrated concurrent loss of MLH1, PMS2, and MSH2, forming a composite dMMR pattern. Although MSH6 expression was preserved, this pattern suggests a more complex MMR dysfunction in the tumor tissue. Notably, all samples with MLH1 or PMS2 abnormalities were from affected family members, and complete loss of MSH2 or MSH6 was not observed, indicating that MMR defects in this family predominantly involve the MLH1–PMS2 pathway.

### 3.3 Microsatellite instability status and STR electropherogram analysis

To further assess the functional status of the MMR system, MSI testing was performed on tumor tissues from four affected family members (II-2, Ⅲ6, Ⅲ11, and Ⅲ21). Multiplex fluorescent PCR combined with capillary electrophoresis was used to evaluate the stability of six loci: BAT25, BAT26, NR21, NR24, NR27, and MONO27. All samples exhibited instability at two or more loci, fulfilling the National Cancer Institute (NCI) criteria for MSI-H classification. Representative electropherograms revealed characteristic alterations in short tandem repeat (STR) fragments, including insertions, deletions, or duplications, typically manifested as double- or triple-peak patterns. For example, in the proband (II-2), NR21, BAT25, and MONO27 loci showed additional or missing fragment signals, resulting in double-band profiles. Sample Ⅲ11 displayed variations at BAT25 and NR21, both presenting double-peak patterns. Sample Ⅲ21 exhibited three distinct fragment lengths (123.5, 127.1, and 131.8 bp) at the NR21 locus, indicating marked instability. Notably, sample Ⅲ6 showed abnormalities at all six loci, with BAT25 demonstrating a classic triple-peak pattern (111.4/114.3/118.9 bp), strongly supporting a functionally defective phenotype. The STR alteration profiles at six loci and their respective MSI classifications are presented in Table 2, with representative electropherogram patterns shown in Figure 2B. Overall, these MSI results were concordant with IHC findings of MLH1 and/or PMS2 loss, further confirming a consistent MMR deficiency in this family.

**TABLE 2:** Summary of STR fragment patterns in MSI testing.

Family ID	BAT25	BAT26	NR21	NR24	NR27	MONO27	MSI status
II2	Double peaks (112/121 bp)	Single	Double peaks (117.9/127.5 bp)	Double peaks (113.6/119.7 bp)	Single	Double peaks (133.2/145.6 bp)	MSI-H
Ⅲ6	Triple peaks (111.4/114.3/118.9 bp)	Double	Double peaks (116.2/122.5 bp)	Double peaks (113.2/119.3 bp)	Double	Triple peaks (130.1/138.4/147.2 bp)	MSI-H
Ⅲ11	Double peaks (112/120 bp)	Single	Double peaks (117.6/127.2 bp)	Single	Single	Single	MSI-H
Ⅲ21	Double peaks (112.8/121.9 bp)	Double	Triple peaks (123.5/127.1/131.8 bp)	Double peaks (113.1/119.8 bp)	Double	Double peaks (134.2/146.0 bp)	MSI-H

Microsatellite status was assessed at six loci using capillary electrophoresis. Fragment shifts were interpreted from electropherograms. Samples with instability at two or more loci were classified as MSI-High (MSI-H).

### 3.4 Molecular confirmation of MLH1 gene alteration and familial Co-segregation analysis

To elucidate the genetic basis underlying the MMR deficiency in this family, peripheral blood from the proband (II-2) was subjected to high-throughput NGS. The results revealed a large heterozygous deletion spanning exon 13 of the *MLH1* gene (c.1410-?_1558+?del), representing a structural variant within the coding region (Figure 2C). This alteration has been cataloged in the ClinVar database and classified as a pathogenic variant, consistent with the American College of Medical Genetics and Genomics (ACMG) criteria for germline pathogenicity. To verify the familial co-segregation of this alteration, peripheral blood samples from five available family members were analyzed using a qPCR assay targeting the *MLH1* exon 13 region. All five individuals exhibited a ∼50% reduction in relative copy number (RQ) compared with a normal genomic DNA control (gDNA1), indicating the presence of a heterozygous deletion and supporting stable inheritance of this variant within the family (Figure 2D). Clinically, among confirmed carriers of this alteration, three individuals had been diagnosed with CRC, and one with endometrioid carcinoma. Tumor tissues from all affected carriers demonstrated dMMR by IHC and MSI-H status, further substantiating the *MLH1*-EX13 Del as the core pathogenic driver in this family.

### 3.5 Identification of differentially expressed proteins by proteomic profiling

**FIGURE 3:**
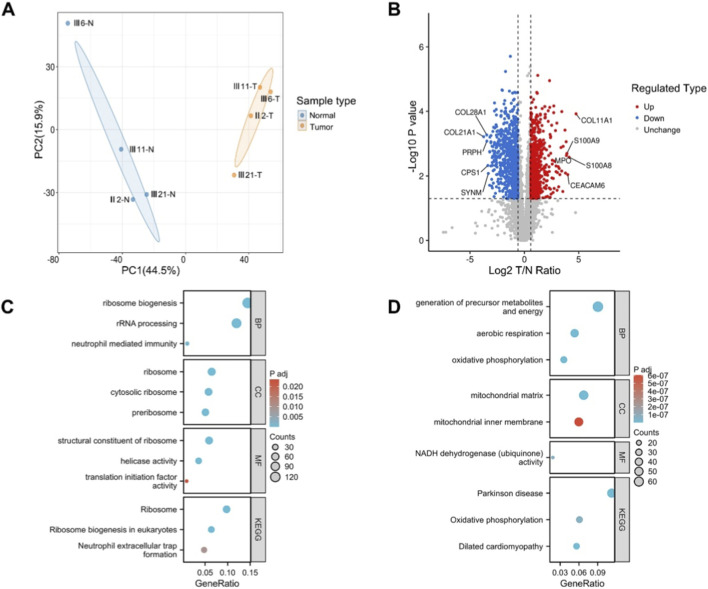
Proteomic profiling reveals distinct expression signatures and pathway alterations in MLH1-deficient colorectal tumors. **(A)** Principal component analysis (PCA) of global proteomic profiles in tumor (T) and matched adjacent normal (N) tissues from four MLH1 mutation carriers, showing clear separation between groups; **(B)** Volcano plot displaying differentially expressed proteins between tumor and normal tissues. Proteins with |log_2_(T/N ratio)| > 1 and adjusted P value <0.05 were considered significant. Key upregulated (e.g., S100A8/A9, CEACAM6) and downregulated (e.g., COL1A1/2) proteins are highlighted; **(C)** GO and KEGG enrichment analysis of upregulated proteins, demonstrating significant enrichment in ribosome biogenesis, rRNA processing, and neutrophil-mediated immunity (BP); cytosolic and preribosome components (CC); helicase activity and translation initiation factor activity (MF); and ribosome-related KEGG pathways; **(D)** GO and KEGG enrichment analysis of downregulated proteins, with major enrichment in mitochondrial energy metabolism processes such as oxidative phosphorylation, aerobic respiration (BP); mitochondrial inner membrane and matrix (CC); NADH dehydrogenase activity (MF); and oxidative phosphorylation and Parkinson’s disease pathways in KEGG.

To further elucidate the functional consequences of the large *MLH1*-EX13 Del at the protein level, we conducted proteomic analysis on paired tumor and adjacent normal mucosal tissues from four mutation-carrying family members (total of eight samples) using the 4D-Fast DIA platform. In total, 7,033 proteins were identified, of which 6,794 were quantified with high confidence. The majority of peptides ranged from 7 to 20 amino acids in length, exhibiting satisfactory sequence coverage. Quantitative depth and inter-sample reproducibility (Pearson’s *r* > 0.95) met the quality requirements for subsequent statistical analyses. Principal component analysis (PCA) demonstrated a clear separation between tumor and adjacent normal tissues in terms of global proteomic profiles, indicating systematic differences between the two groups (Figure 3A). Using a significance threshold of *P* < 0.05 and a fold-change cut-off of >1.5 (upregulated) or <1/1.5 (downregulated), a total of 1,681 proteins were identified as significantly differentially expressed, including 882 upregulated and 799 downregulated proteins (Figure 3B).

### 3.6 Functional annotation and pathway enrichment of differentially expressed proteins

To explore the potential biological implications of MLH1-deficient tumors, Gene GO and KEGG pathway enrichment analyses were performed for both upregulated and downregulated protein sets. Upregulated proteins were predominantly enriched in pathways related to protein biosynthesis and immune responses. GO biological process analysis revealed significant involvement in “ribosome biogenesis” and “rRNA processing”. At the cellular component level, these proteins were mainly localized to the “ribosome” and “cytosolic ribosome,” while molecular function analysis highlighted enrichment in “structural constituent of ribosome” and “translation initiation factor activity.” KEGG analysis further identified “Ribosome” and “Eukaryotic ribosome biogenesis” as the most significantly upregulated pathways. Additionally, immune activation–related pathways such as “neutrophil-mediated immunity” and “neutrophil extracellular trap formation” were significantly enriched, suggesting an active inflammatory and immune infiltration profile in these tumors (Figure 3C).In contrast, downregulated proteins were mainly associated with mitochondrial energy metabolism. GO biological process terms were enriched for “oxidative phosphorylation,” “aerobic respiration,” and “generation of precursor metabolites and energy.” These proteins were primarily localized to the “mitochondrial matrix” and “mitochondrial inner membrane,” with molecular functions enriched in “NADH dehydrogenase (ubiquinone) activity.” KEGG pathway analysis similarly indicated downregulation of oxidative phosphorylation and other mitochondrial function–related pathways, such as “Parkinson’s disease,” suggesting metabolic reprogramming with potential mitochondrial functional suppression in tumor cells (Figure 3D).

### 3.7 Proteomic evidence of dual features: metabolic reprogramming and immune activation

**FIGURE 4:**
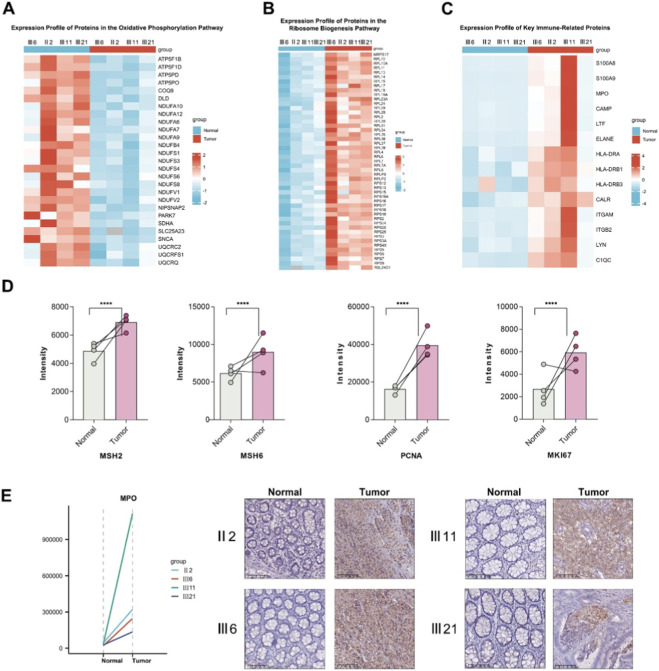
Expression profiling of key pathway proteins in MLH1-deficient tumors. **(A)** Heatmap showing the expression levels of proteins involved in the oxidative phosphorylation (OxPhos) pathway. Most core components of mitochondrial electron transport chain complexes I–V are significantly downregulated in tumor samples; **(B)** Heatmap illustrating the upregulation of ribosomal proteins in the ribosome biogenesis pathway, including both small (RPS) and large (RPL) subunits; **(C)** Heatmap of selected immune-related proteins. Significant upregulation is observed in neutrophil markers (S100A8, S100A9, MPO, ELANE), antigen presentation molecules (HLA family), and immune modulators (ITGB2, LTF); **(D)** Quantitative bar plots comparing the expression intensity of MSH2, MSH6 (core MMR proteins), and proliferation markers PCNA and MKI67 between tumor and paired normal tissues. All four proteins are significantly upregulated in tumor tissues (****P < 0.0001); **(E)** Validation of MPO expression by proteomics and immunohistochemistry (IHC). The left panel shows the quantitative proteomic intensity of MPO in four paired tumor (T) and adjacent normal (N) tissues from family members (II-2, III-6, III-11, and III-21). The right panel displays representative IHC staining, demonstrating markedly increased cytoplasmic MPO expression in tumor tissues, consistent with proteomic findings. Scale bars: 100 μm.

Building on the enrichment analysis results indicating that differentially expressed proteins were primarily involved in metabolic remodeling and immune response pathways, we further selected representative proteins from these pathways and constructed heatmaps to validate their expression patterns. The selected pathways included oxidative phosphorylation (as a proxy for mitochondrial energy metabolism), ribosome biogenesis (reflecting cellular protein synthesis and proliferative capacity), and immune responses (representing tumor microenvironment activity) (Figures 4A–C).

At the metabolic level, key proteins in the oxidative phosphorylation pathway-such as ATP5F1B, NDUFA9, and UQCRC2—were consistently downregulated across all tumor samples, covering multiple core subunits of the mitochondrial respiratory chain complexes (Figure 4A). This indicates suppression of the mitochondrial energy production system, consistent with the classic “Warburg effect,” whereby tumor cells downregulate efficient aerobic metabolism and instead rely on glycolysis to sustain rapid proliferation and provide biosynthetic intermediates. In contrast, ribosome-associated proteins were significantly upregulated in tumor tissues, particularly members of the RPL and RPS families (Figure 4B). This suggests a marked activation of protein synthesis machinery, which may serve as an adaptive mechanism to support high transcriptional activity and accelerated growth of tumor cells.

At the immune response level, the heatmap revealed pronounced upregulation of proteins involved in inflammatory signaling and antigen presentation (Figure 4C). Myeloid cell–associated markers such as S100A8/A9, MPO, and ELANE were highly expressed, indicating robust neutrophil infiltration. In addition, enhanced expression of proteins related to antigen processing and immune activation, including HLA-DRA, ITGAM, and C1QC, reflected a strong inflammatory phenotype within the tumor tissue. Together, these findings demonstrate that dMMR tumors exhibit a markedly activated immune state, characterized by a “hot tumor” phenotype dominated by neutrophil infiltration.

### 3.8 Validation of key proteins and multi-omics integration analysis

To validate the accuracy of the proteomic data and to integrate upstream genetic alterations with clinicopathological findings, we focused on the expression profiles of DNA MMR pathway proteins and tumor proliferation markers (Figures 4D,E).

In the MMR pathway, proteomic analysis revealed loss of MLH1 and PMS2 expression, whereas MSH2 and MSH6 were markedly upregulated, constituting a typical dMMR pattern of “intact recognition but defective repair.” This phenomenon may represent a compensatory response. IHC results also confirmed the loss of MLH1 and PMS2 expression, in line with the proteomic findings (Figure 2A). At the functional level, NGS of the proband’s peripheral blood identified a large heterozygous deletion in exon 13 of the MLH1 gene (EX13 Del). This pathogenic germline variant leads to the absence of MLH1 protein and the secondary degradation of PMS2 due to their chaperone-dependent stability, thereby providing the molecular basis for MMR dysfunction and MSI-H phenotype observed in this family.

To further validate immune-related and proliferative alterations revealed by proteomics, we performed IHC for MPO and reviewed archival Ki-67 staining results. Consistent with the proteomic data, MPO showed markedly increased cytoplasmic expression in tumor tissues compared with adjacent normal mucosa, confirming enhanced neutrophil-mediated immune activation (Figure 4E). Additionally, Ki-67 IHC results retrieved from the same cases (Supplementary Figure S2) demonstrated strong nuclear positivity, consistent with the upregulation of proliferation-associated proteins (PCNA and MKI67) detected in the proteomic analysis.

Collectively, these multi-omics findings establish a coherent molecular cascade from the MLH1 exon 13 deletion to the loss of MMR protein stability, activation of compensatory pathways, and enhanced proliferative and inflammatory phenotypes in this Lynch syndrome family, reinforcing the pathogenic role of this rare germline variant.

## 4 Discussion

In this study, we systematically analyzed a three-generation Chinese family with LS carrying a large heterozygous deletion of exon 13 in the *MLH1* gene (EX13 Del). The pathogenicity of this variant was validated by qPCR in multiple family members. By integrating clinical data, pathological examination, MMR IHC, and MSI testing, we reconstructed the molecular cascade from germline *MLH1* deficiency to the functional phenotype. Previous reports of *MLH1*-EX13 Del have been limited to sporadic case descriptions (McVety et al., 2006; Chong et al., 2009; van der Klift et al., 2005; Liu et al., 1995), lacking systematic investigation of its downstream biological consequences. The novelty of our work lies in the first application of unbiased quantitative proteomics to comprehensively characterize the tumor molecular features driven by this specific LGR. This integrative approach establishes a new paradigm for elucidating the pathogenic mechanisms of CRC and provides valuable insights into precision oncology.

Compared with previous studies, several international efforts have attempted to characterize the molecular features of dMMR colorectal cancers using multi-omics approaches. The TCGA project first integrated genomic, transcriptomic, and methylation data to delineate the distinct mutation spectra, signalling activation, and immune infiltration patterns of MSI-H tumors (Cancer Genome Atlas, 2012). While these large-scale studies provided important subtype-level insights, they were largely based on heterogeneous cohorts and did not resolve the downstream functional consequences of specific familial pathogenic variants. Additional investigations, including a metabolomics study in Lynch syndrome carriers and a comprehensive review on hereditary colorectal cancer syndromes, have expanded our understanding of LS (Jokela et al., 2024; Nolano et al., 2022). Nevertheless, these analyses rarely extended to proteomic characterization or explored how germline mutations remodel cellular metabolism and the immune microenvironment. In contrast, our study not only validated the pathogenicity of *MLH1-EX13 Del* in a multi-member Chinese family but also, for the first time, applied unbiased quantitative proteomics to comprehensively annotate the downstream functional landscape of this LGR-driven tumor. This family-based multi-omics strategy bridges the gap between broad cohort-level molecular profiling and the precise mechanistic understanding of inherited *MLH1*-deficient tumors.

At the molecular mechanistic level, we integrated existing epidemiological evidence with our study data to elucidate the pathobiological relevance of the MLH1-EX13 Del variant. MLH1 is one of the core genes of the MMR pathway, encoding a protein that forms the MutLα complex with PMS2, which participates in the excision and repair of DNA mismatches. Large genomic rearrangements (LGRs) represent an important pathogenic type of MLH1 variants, yet such alterations may be underestimated in clinical practice due to the limitations of conventional NGS in detecting structural variants. Multicenter studies have demonstrated that incorporating MLPA into Lynch syndrome screening can substantially improve the detection of LGRs, revealing additional pathogenic variants that might otherwise be missed by routine NGS testing (Feng et al., 2024). In our study, NGS identified and qPCR confirmed a heterozygous large deletion in exon 13 of MLH1, which was further corroborated by proteomic and immunohistochemical analyses showing complete loss of MLH1 and PMS2 expression-constituting a direct evidence chain from gene to protein. This finding is fully consistent with the known molecular mechanism whereby MLH1 deficiency results in degradation of its partner protein PMS2. Notably, proteomic data also revealed upregulation of MSH2 and MSH6. Previous studies have shown that even in the absence of MLH1/PMS2, the expression of MSH2 and MSH6 is generally preserved (Reitsam et al., 2022), indicating that their stability is not dependent on the MutLα complex. Our results further suggest that such upregulation may represent a compensatory regulatory mechanism, whereby cells increase protein levels of the mismatch recognition module (MSH2–MSH6 complex) to partially offset the loss of the repair module (MLH1–PMS2 complex). However, because the repair function of MutLα is irreplaceable, this mechanism cannot prevent the development of the dMMR/MSI-H phenotype, while also providing new evidence for understanding the functional interplay within the MMR pathway.

At the functional level, this study revealed marked alterations in two key directions-metabolism and immunity-in MLH1-deficient tumors. At the proteomic level, we for the first time directly linked the dMMR state caused by a specific germline large deletion in MLH1 with systemic suppression of oxidative phosphorylation and robust activation of ribosome biogenesis. OXPHOS-related proteins (e.g., ATP5F1B, NDUFA9, UQCRC2) were broadly downregulated in tumor tissues, spanning multiple core subunits of the five complexes of the mitochondrial electron transport chain, suggesting impaired mitochondrial energy production. In contrast, numerous structural proteins of the ribosomal 40S and 60S subunits (RPS and RPL families) were significantly upregulated, reflecting strong activation of the protein synthesis machinery. This molecular signature not only corresponds to the proteomic manifestation of the Warburg effect but also aligns with the notion that aberrant activation of ribosome biogenesis and translational processes plays an important biological role in multiple cancers and may represent a potential therapeutic target (Pelletier et al., 2018; Penzo et al., 2019). In CRC specifically, systematic reviews have already summarized related evidence and highlighted the potential druggability of these pathways (Nait et al., 2020). Together, these findings highlight that metabolic reprogramming, particularly the suppression of mitochondrial OXPHOS alongside hyperactivation of ribosome biogenesis, constitutes a core molecular hallmark of MLH1-deficient tumors, warranting further investigation into its mechanistic basis and therapeutic vulnerabilities.

In terms of immune characteristics, these tumors exhibited a pronounced inflammatory-immune phenotype. Enrichment analysis indicated upregulation of “neutrophil-mediated immunity” pathways, accompanied by high expression of neutrophil markers (S100A8/A9, MPO, ELANE) and antigen-presenting molecules (HLA-DRA, ITGAM, C1QC), suggestive of a “hot tumor” phenotype. Notably, the marked upregulation of S100A8/A9 not only serves as a hallmark of neutrophil infiltration and a pro-inflammatory tumor microenvironment (Wu et al., 2022), but is also closely linked to the activation of immune-related pathways. The observed robust neutrophil infiltration (high MPO, S100A8/A9, ELANE) raises important questions about its functional role in the context of immunotherapy. Tumor-associated neutrophils (TANs) can exhibit a dual role, adopting either an anti-tumor N1-like phenotype or a pro-tumor, immunosuppressive N2-like phenotype, often dependent on the cytokine context (e.g., IFN-γpromoting N1, versus TGF-βdriving N2) (Huang et al., 2024). While our bulk proteomic data cannot definitively resolve this polarization, the overall context of dMMR/MSI-H tumors is critical. These tumors are known to be highly immunogenic and characterized by a T-cell inflamed phenotype with strong IFN-γ-related gene expression (Chen et al., 2022), which is known to favor N1 polarization. Therefore, it is plausible that the robust inflammatory milieu we detected—characterized by high S100A8/A9 and upregulated antigen presentation machinery (e.g., HLA-DRA)—corresponds to an activated, N1-like phenotype. This N1-like activity would contribute to the overall “hot tumor” state, which supports the known favorable response of dMMR/MSI-H tumors to immune checkpoint inhibitors (Le et al., 2015; Andre et al., 2020). Distinguishing the precise N1/N2 balance within this specific MLH1-EX13 Del context remains a critical area for future investigation using techniques like spatial proteomics or single-cell analysis. Collectively, these findings reinforce that the inflammatory, N1-like neutrophil activity may contribute to the favorable immunotherapy response observed in MSI-H/dMMR tumors. Importantly, consistent with our findings, a multi-omics analysis of colorectal medullary carcinoma (MeC) also identified a pattern of significant immune pathway upregulation coupled with metabolic pathway downregulation in the MSI-H/dMMR subtype (Liu et al., 2025), further substantiating the interplay between metabolic suppression and immune activation in this tumor type.

These findings carry significant translational implications. The dual signature of metabolic suppression and immune activation offers a strong rationale for novel therapeutic strategies. The systematic downregulation of oxidative phosphorylation (OXPHOS) subunits across the mitochondrial respiratory chain underscores a shift toward glycolysis and a distinct metabolic vulnerability (Qin et al., 2023). Meanwhile, the observed upregulation of neutrophil-related markers (S100A8/A9, MPO) and antigen-presentation machinery (HLA-DRA, ITGAM) reflects an inflamed tumor microenvironment typical of “hot tumors” and supports immune checkpoint inhibitor sensitivity (Zheng et al., 2022). Together, these features suggest that combining metabolic intervention (e.g., glycolysis or mitochondrial inhibitors) with immunotherapy may provide synergistic benefits in MLH1-deficient Lynch syndrome-associated colorectal cancer and deserve further pre-clinical and clinical exploration. The concordant upregulation of MPO and Ki67 at both proteomic and IHC levels provides independent validation of the DIA-based findings and strengthens the evidence supporting the dual molecular hallmark of metabolic suppression and immune activation in MLH1-deficient tumors.

A key question raised by this study is whether the observed “metabolic suppression–immune activation” phenotype is unique to the MLH1-EX13 Del variant or represents a general feature of MLH1-deficient tumors. While our analysis is based on a single germline large genomic rearrangement, our findings are consistent with growing evidence that this dual phenotype constitutes a shared molecular hallmark of the MSI-H/dMMR subtype. Recent reviews have established that the metabolic shift from oxidative phosphorylation to glycolysis (the Warburg effect) plays a central role in shaping the tumor immune microenvironment and influences the response to immune checkpoint therapy (Nicolini and Ferrari, 2024; Bowen et al., 2025). Furthermore, large-scale proteogenomic and proteo-metabolomic analyses comparing MSI-H and MSS tumors have confirmed that MSI-H tumors are characterized by suppressed mitochondrial metabolism together with enhanced immune infiltration and activation (Vasaikar et al., 2019; Rashid et al., 2019). These findings collectively indicate that the metabolic–immune dual signature identified in our MLH1-EX13 Del case reflects a common downstream consequence of dMMR-driven tumor evolution, rather than a variant-specific effect.

**FIGURE 5:**
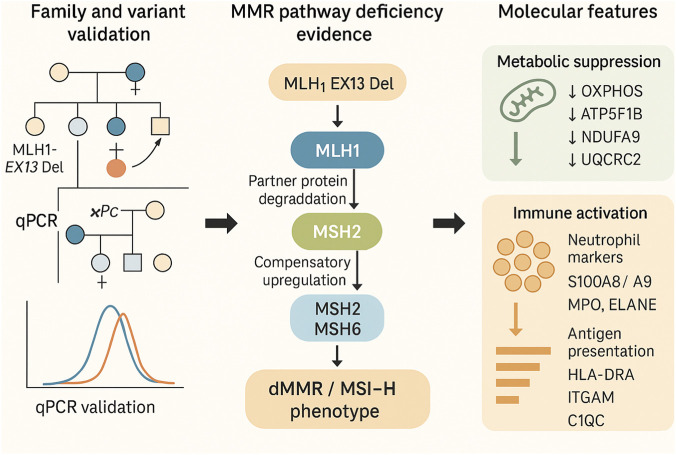
Mechanistic model of MLH1-EX13 Del-driven CRC. Germline *MLH1*-EX13 Del leads to MMR deficiency and MSI-H phenotype, accompanied by suppressed oxidative phosphorylation, activated ribosome biogenesis, and a pro-inflammatory immune microenvironment, together shaping the dual hallmark of “metabolic suppression-immune activation.”

In summary, this study provides a comprehensive molecular characterization of a multi-generation Lynch syndrome family carrying the pathogenic *MLH1*-EX13 deletion, establishing its causative role through integrated genetic, pathological, and proteomic evidence. Beyond confirming the dMMR/MSI-H phenotype, our proteomic findings delineate a distinct dual molecular hallmark of “metabolic suppression and immune activation,” reflecting both impaired mitochondrial energy metabolism and an inflamed tumor microenvironment. These results not only expand the current understanding of *MLH1*-deficient colorectal cancer but also highlight the translational potential of targeting mitochondrial metabolism alongside immune checkpoint pathways as a combinatorial therapeutic strategy. Clinically, this work reinforces the necessity of comprehensive genetic testing that includes large genomic rearrangements for accurate diagnosis, genetic counseling, and proactive surveillance of at-risk family members. Despite these insights, several limitations should be acknowledged. The sample size was confined to a single family, and proteomic analyses were performed on only four pairs of matched tumor and adjacent tissues. Thus, validation in larger, multi-center cohorts and functional confirmation using cellular, organoid, or animal models will be essential to establish the mechanistic links between *MLH1* loss, metabolic reprogramming, and immune modulation. Future studies integrating multi-omics approaches such as transcriptomics, metabolomics, and spatially resolved proteogenomics are warranted to further elucidate *MLH1*-deficiency-driven tumor evolution at both cellular and microenvironmental levels. Moreover, advances in intestinal organoid systems now provide powerful experimental models for investigating colorectal tumor biology and validating multi-omics findings (Zhang and Wang, 2025). To summarize our conceptual framework, Figure 5 presents an integrative schematic model illustrating the pathogenic cascade from germline *MLH1* deficiency to proteomic remodeling, leading to the dual hallmark of metabolic suppression and immune activation.

## 5 Conclusion

This study systematically demonstrated the pathogenicity of MLH1 exon 13 heterozygous deletion (EX13 Del) in a three-generation Chinese Lynch syndrome family, establishing a direct evidence chain from germline deficiency to proteomic phenotype. We identified a dual hallmark of MLH1-deficient colorectal cancer- “metabolic suppression-immune activation”-that expands the mechanistic understanding of mismatch repair deficiency and provides a rationale for exploring combined therapeutic strategies targeting mitochondrial metabolic vulnerabilities and immune checkpoint pathways. Clinically, our findings emphasize the necessity of incorporating structural variant detection into genetic counseling and surveillance protocols. Validation in larger cohorts and functional models is warranted to confirm these insights and facilitate their translation into precision oncology practice.

## Data Availability

The mass spectrometry proteomics data generated in this study have been deposited to the ProteomeXchange Consortium (https://proteomecentral.proteomexchange.org) via the iProX partner repository (https://www.iprox.cn/page/project.html?id=IPX00146180000) under the dataset identifier PXD071799. All data are publicly available.

## References

[B1] Abu-Ghazaleh N. Kaushik V. Gorelik A. Jenkins M. Macrae F. (2022). Worldwide prevalence of Lynch syndrome in patients with colorectal cancer: systematic review and meta-analysis. Genet. Med. 24 (5), 971–985. 10.1016/j.gim.2022.01.014 35177335

[B2] Andre T. Shiu K. K. Kim T. W. Jensen B. V. Jensen L. H. Punt C. (2020). Pembrolizumab in microsatellite-instability-high advanced colorectal cancer. N. Engl. J. Med. 383 (23), 2207–2218. 10.1056/NEJMoa2017699 33264544

[B3] Bowen M. B. Melendez B. Zhang Q. Moreno D. Peralta L. Chan W. K. (2025). Mitochondrial defects and metabolic vulnerabilities in Lynch syndrome-associated MSH2-deficient endometrial cancer. JCI Insight 10 (6), e185946. 10.1172/jci.insight.185946 39964762 PMC11949016

[B4] Cancer Genome Atlas N. (2012). Comprehensive molecular characterization of human colon and rectal cancer. Nature 487 (7407), 330–337. 10.1038/nature11252 22810696 PMC3401966

[B5] Chen Q. Yin H. Liu S. Shoucair S. Ding N. Ji Y. (2022). Prognostic value of tumor-associated N1/N2 neutrophil plasticity in patients following radical resection of pancreas ductal adenocarcinoma. J. Immunother. Cancer 10 (12), e005798. 10.1136/jitc-2022-005798 36600557 PMC9730407

[B6] Chong G. Jarry J. Marcus V. Thiffault I. Winocour S. Monczak Y. (2009). High frequency of exon deletions and putative founder effects in French Canadian Lynch syndrome families. Hum. Mutat. 30 (8), E797–E812. 10.1002/humu.21056 19459153

[B7] Fanale D. Corsini L. R. Brando C. Dimino A. Filorizzo C. Magrin L. (2022). Impact of different selection approaches for identifying Lynch syndrome-related colorectal cancer patients: unity is strength. Front. Oncol. 12, 827822. 10.3389/fonc.2022.827822 35223509 PMC8864140

[B8] Feng X. Yao Q. Xu Y. Zhang J. Jia L. Wang Q. (2024). Approaches for Lynch syndrome screening and characteristics of subtypes with mismatch repair deficiency in patients with colorectal carcinoma. Int. J. Cancer 155 (10), 1780–1791. 10.1002/ijc.35085 39109916

[B9] Huang J. Stinnett V. Jiang L. Chen S. Rodriguez F. Gocke C. D. (2022). Lynch syndrome caused by a novel deletion of the promoter and exons 1-13 of MLH1 gene. Cancer Genet. 262-263, 91–94. 10.1016/j.cancergen.2022.01.005 35149321

[B10] Huang X. Nepovimova E. Adam V. Sivak L. Heger Z. Valko M. (2024). Neutrophils in cancer immunotherapy: friends or foes? Mol. Cancer 23 (1), 107. 10.1186/s12943-024-02004-z 38760815 PMC11102125

[B11] Idos G. Valle L. Syndrome L. (1993). In: GeneReviews((R)) edn. Edited by Adam M. P. Feldman J. Mirzaa G. M. Pagon R. A. Wallace S. E. Amemiya A. Seattle (WA).

[B12] Jokela T. A. Karppinen J. E. Karkkainen M. Mecklin J. P. Walker S. Seppala T. T. (2024). Circulating metabolome landscape in Lynch syndrome. Cancer Metab. 12 (1), 4. 10.1186/s40170-024-00331-9 38317210 PMC10840166

[B13] Le D. T. Uram J. N. Wang H. Bartlett B. R. Kemberling H. Eyring A. D. (2015). PD-1 blockade in tumors with mismatch-repair deficiency. N. Engl. J. Med. 372 (26), 2509–2520. 10.1056/NEJMoa1500596 26028255 PMC4481136

[B14] Liu B. Farrington S. M. Petersen G. M. Hamilton S. R. Parsons R. Papadopoulos N. (1995). Genetic instability occurs in the majority of young patients with colorectal cancer. Nat. Med. 1 (4), 348–352. 10.1038/nm0495-348 7585065

[B15] Liu C. Zou H. Ruan Y. Fang L. Wang B. Cui L. (2025). Multiomics reveals the immunologic features and the immune checkpoint blockade potential of colorectal medullary carcinoma. Clin. Cancer Res. 31 (4), 773–786. 10.1158/1078-0432.CCR-24-2505 39651997 PMC11831109

[B16] McVety S. Li L. Thiffault I. Gordon P. H. Macnamara E. Wong N. (2006). The value of multi-modal gene screening in HNPCC in Quebec: three mutations in mismatch repair genes that would have not been correctly identified by genomic DNA sequencing alone. Fam. Cancer 5 (1), 21–28. 10.1007/s10689-005-2572-6 16528605

[B17] Nait S. S. Marcel V. Fenouil T. Catez F. Saurin J. C. Bouvet P. (2020). Ribosome biogenesis alterations in colorectal cancer. Cells 9 (11), 2361. 10.3390/cells9112361 33120992 PMC7693311

[B18] Nicolini A. Ferrari P. (2024). Involvement of tumor immune microenvironment metabolic reprogramming in colorectal cancer progression, immune escape, and response to immunotherapy. Front. Immunol. 15, 1353787. 10.3389/fimmu.2024.1353787 39119332 PMC11306065

[B19] Nolano A. Medugno A. Trombetti S. Liccardo R. De Rosa M. Izzo P. (2022). Hereditary colorectal cancer: state of the art in Lynch syndrome. Cancers (Basel) 15 (1), 75. 10.3390/cancers15010075 36612072 PMC9817772

[B20] Pelletier J. Thomas G. Volarevic S. (2018). Ribosome biogenesis in cancer: new players and therapeutic avenues. Nat. Rev. Cancer 18 (1), 51–63. 10.1038/nrc.2017.104 29192214

[B21] Peltomaki P. Nystrom M. Mecklin J. P. Seppala T. T. (2023). Lynch syndrome genetics and clinical implications. Gastroenterology 164 (5), 783–799. 10.1053/j.gastro.2022.08.058 36706841

[B22] Penzo M. Montanaro L. Trere D. Derenzini M. (2019). The ribosome biogenesis-cancer connection. Cells 8 (1), 55. 10.3390/cells8010055 30650663 PMC6356843

[B23] Qin R. Huang Y. Yao Y. Wang L. Zhang Z. Huang W. (2023). The role and molecular mechanism of metabolic reprogramming of colorectal cancer by UBR5 through PYK2 regulation of OXPHOS expression study. J. Biochem. Mol. Toxicol. 37 (8), e23376. 10.1002/jbt.23376 37098808

[B24] Rashid S. Freitas M. O. Cucchi D. Bridge G. Yao Z. Gay L. (2019). MLH1 deficiency leads to deregulated mitochondrial metabolism. Cell Death Dis. 10 (11), 795. 10.1038/s41419-019-2018-y 31641109 PMC6805956

[B25] Reitsam N. G. Markl B. Dintner S. Waidhauser J. Vlasenko D. Grosser B. (2022). Concurrent loss of MLH1, PMS2 and MSH6 immunoexpression in digestive system cancers indicating a widespread dysregulation in DNA repair processes. Front. Oncol. 12, 1019798. 10.3389/fonc.2022.1019798 36387226 PMC9643848

[B26] Samowitz W. S. (2015). Evaluation of colorectal cancers for Lynch syndrome: practical molecular diagnostics for surgical pathologists. Mod. Pathol. 28 (Suppl. 1), S109–S113. 10.1038/modpathol.2014.127 25560596

[B27] van der Klift H. Wijnen J. Wagner A. Verkuilen P. Tops C. Otway R. (2005). Molecular characterization of the spectrum of genomic deletions in the mismatch repair genes MSH2, MLH1, MSH6, and PMS2 responsible for hereditary nonpolyposis colorectal cancer (HNPCC). Genes Chromosom. Cancer 44 (2), 123–138. 10.1002/gcc.20219 15942939

[B28] Vasaikar S. Huang C. Wang X. Petyuk V. A. Savage S. R. Wen B. (2019). Proteogenomic analysis of human Colon cancer reveals new therapeutic opportunities. Cell 177 (4), 1035–1049 e1019. 10.1016/j.cell.2019.03.030 31031003 PMC6768830

[B29] Witt D. Faust U. Strobl-Wildemann G. Sturm M. Buchert R. Zuleger T. (2023). Genome sequencing identifies complex structural MLH1 variant in unsolved Lynch syndrome. Mol. Genet. Genomic Med. 11 (6), e2151. 10.1002/mgg3.2151 36760167 PMC10265068

[B30] Wu Z. Jiang D. Huang X. Cai M. Yuan K. Huang P. (2022). S100A8 as a promising biomarker and oncogenic immune protein in the tumor microenvironment: an integrative pancancer analysis. J. Oncol. 2022, 6947652. 10.1155/2022/6947652 35646116 PMC9132702

[B31] Zhang D. D. Wang P. Y. (2025). Intestinal stem cells (ISCs): iscs-derived organoids for disease modeling and therapy. Eur. Cells Mater 50, 84–86. 10.22203/ecm.v050a05

[B32] Zheng W. Wu J. Peng Y. Sun J. Cheng P. Huang Q. (2022). Tumor-Associated neutrophils in colorectal cancer development, progression and immunotherapy. Cancers (Basel) 14 (19), 4755. 10.3390/cancers14194755 36230676 PMC9563115

