# Supplementary material for: Integrative proteomics reveals mitochondrial and immune signatures of MLH1 exon 13 deletion in Lynch syndrome–associated colorectal cancer
Source: Front Mol Biosci. 2025 Dec 17;12:1722111. doi: 10.3389/fmolb.2025.1722111 (PMC12753366; doi:10.3389/fmolb.2025.1722111)
Supplement: Supplementary file 1 [file Supplementaryfile1.docx]

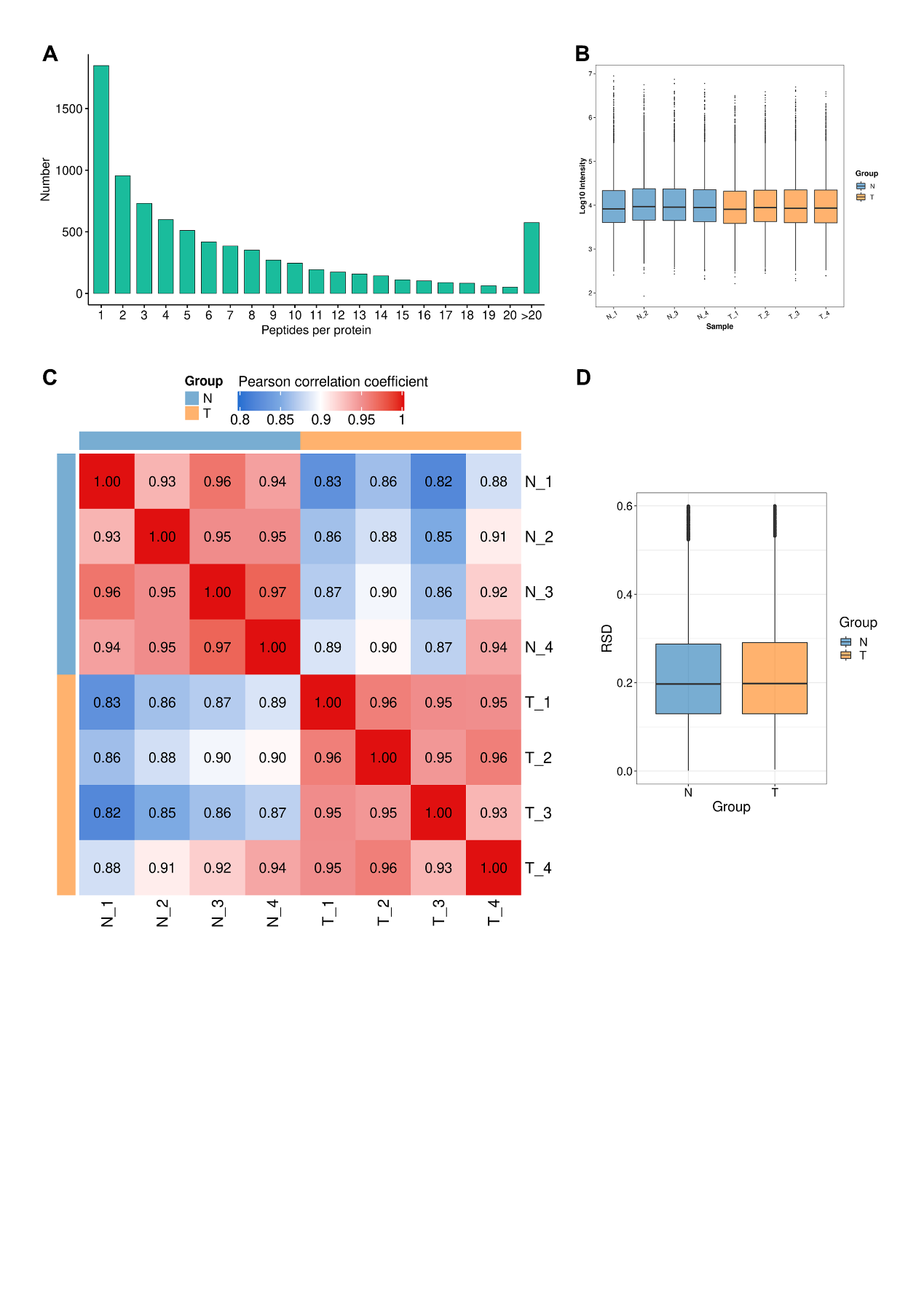


**Figure S1. Quality control of DIA-based proteomic data.** **(A)** Peptide count distribution showing that most proteins were identified by 1–10 peptides, confirming adequate peptide coverage. **(B)** Boxplot of log₂ intensities demonstrating consistent normalization across all tumor (T) and normal (N) samples. **(C)** Pearson correlation heatmap showing strong reproducibility among biological replicates (r > 0.9). **(D)** Relative standard deviation (RSD) distribution of quantified proteins indicating high stability of protein quantification (majority < 0.2).


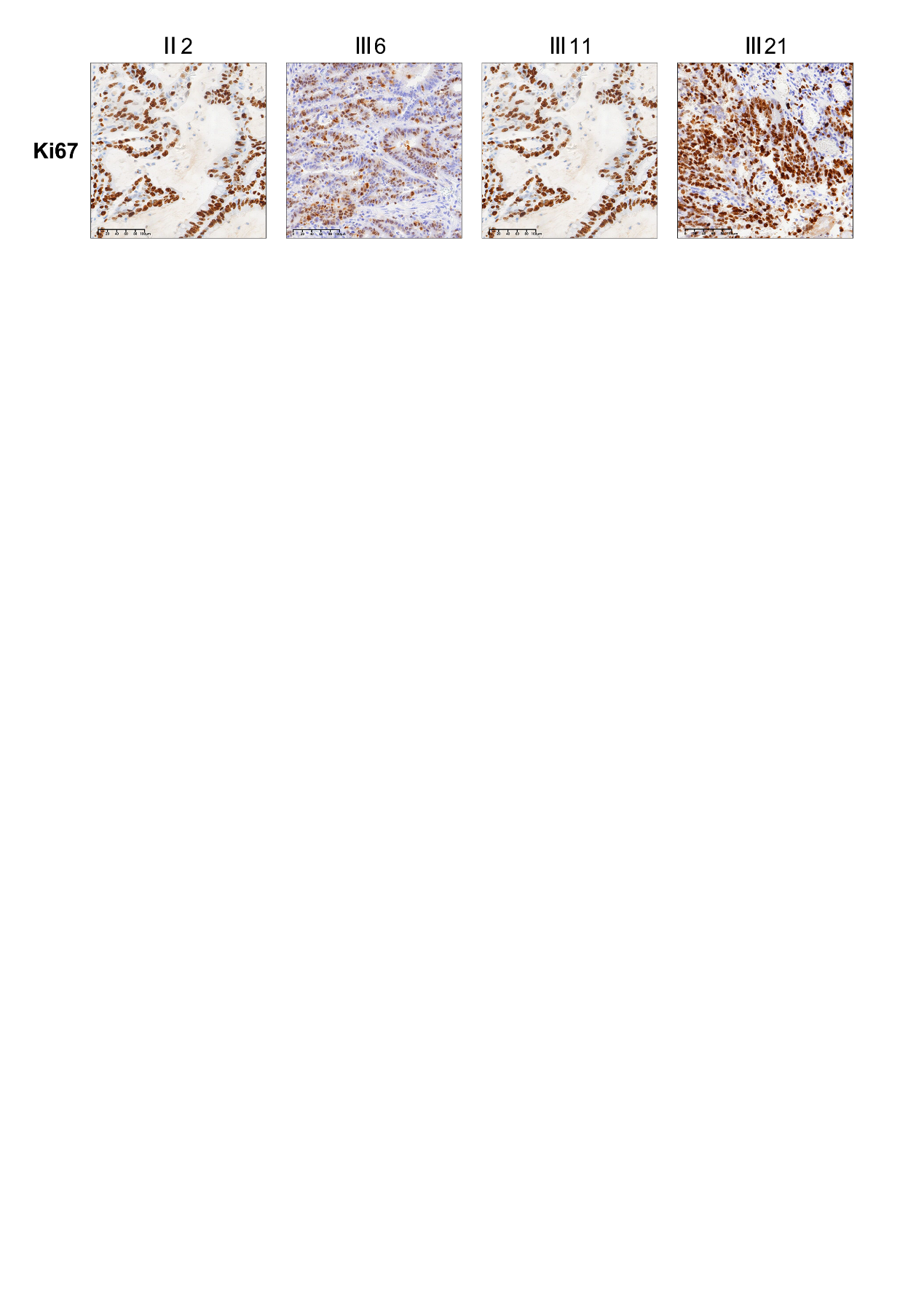


**Figure S2. Immunohistochemical staining of Ki67 in tumor tissues.** Representative IHC images showing nuclear Ki67 expression in tumor tissues from family members (II-2, III-6, III-11, and III-21). Strong nuclear positivity was observed across all cases, indicating high proliferative activity consistent with proteomic upregulation of PCNA and MKI67. Scale bars: 100 μm.
